# Serotypes, virulence genes and intimin types of Shiga toxin (verocytotoxin)-producing *Escherichia coli *isolates from minced beef in Lugo (Spain) from 1995 through 2003

**DOI:** 10.1186/1471-2180-7-13

**Published:** 2007-03-01

**Authors:** Azucena Mora, Miguel Blanco, Jesús E Blanco, Ghizlane Dahbi, Cecilia López, Paula Justel, María Pilar Alonso, Aurora Echeita, María Isabel Bernárdez, Enrique A González, Jorge Blanco

**Affiliations:** 1 Laboratorio de Referencia de *E. coli *(LREC), Departamento de Microbioloxía e Parasitoloxía, Universidade de Santiago de Compostela (USC), 27002 Lugo, Spain; 2 Unidade de Microbioloxía Clínica, Complexo Hospitalario Xeral-Calde, 27004 Lugo, Spain; 3 Laboratorio de Bacteriología, Centro Nacional de Microbiología, Instituto de Salud Carlos III, Madrid, Spain; 4 Deceased

## Abstract

**Background:**

Shiga toxin-producing *Escherichia coli *(STEC) have emerged as pathogens that can cause food-borne infections and severe and potentially fatal illnesses in humans, such as haemorrhagic colitis (HC) and haemolytic uraemic syndrome (HUS). In Spain, like in many other countries, STEC strains have been frequently isolated from ruminants, and represent a significant cause of sporadic cases of human infection. In view of the lack of data on STEC isolated from food in Spain, the objectives of this study were to determine the level of microbiological contamination and the prevalence of STEC O157:H7 and non-O157 in a large sampling of minced beef collected from 30 local stores in Lugo city between 1995 and 2003. Also to establish if those STEC isolated from food possessed the same virulence profiles as STEC strains causing human infections.

**Results:**

STEC were detected in 95 (12%) of the 785 minced beef samples tested. STEC O157:H7 was isolated from eight (1.0%) samples and non-O157 STEC from 90 (11%) samples. Ninety-six STEC isolates were further characterized by PCR and serotyping. PCR showed that 28 (29%) isolates carried *stx*_1 _genes, 49 (51%) possessed *stx*_2 _genes, and 19 (20%) both *stx*_1 _and *stx*_2_. Enterohemolysin (*ehxA*) and intimin (*eae*) virulence genes were detected in 43 (45%) and in 25 (26%) of the isolates, respectively. Typing of the *eae *variants detected four types: γ1 (nine isolates), β1 (eight isolates), ε1 (three isolates), and θ (two isolates). The majority (68%) of STEC isolates belonged to serotypes previously detected in human STEC and 38% to serotypes associated with STEC isolated from patients with HUS. Ten new serotypes not previously described in raw beef products were also detected. The highly virulent seropathotypes O26:H11 *stx*_1 _*eae*-β1, O157:H7 *stx*_1_*stx*_2 _*eae*-γ1 and O157:H7 *stx*_2_*eae*-γ1, which are the most frequently observed among STEC causing human infections in Spain, were detected in 10 of the 96 STEC isolates. Furthermore, phage typing of STEC O157:H7 isolates showed that the majority (seven of eight isolates) belonged to the main phage types previously detected in STEC O157:H7 strains associated with severe human illnesses.

**Conclusion:**

The results of this study do not differ greatly from those reported in other countries with regard to prevalence of O157 and non-O157 STEC in minced beef. As we suspected, serotypes different from O157:H7 also play an important role in food contamination in Spain, including the highly virulent seropathotype O26:H11 *stx*_1 _*eae*-β1. Thus, our data confirm minced beef in the city of Lugo as vehicles of highly pathogenic STEC. This requires that control measures to be introduced and implemented to increase the safety of minced beef.

## Background

Shiga toxin-producing *Escherichia coli *(STEC), also called verocytotoxin-producing *E. coli *(VTEC), have emerged as pathogens that can cause food-borne infections and severe and potentially fatal illnesses in humans, such as haemorrhagic colitis (HC) and haemolytic uraemic syndrome (HUS). STEC strains that cause human infections belong to a large number of O:H serotypes. Certain STEC strains belonging to serotypes O26:H11, O103:H2, O111:H8, O145:H28, and O157:H7 have been more frequently isolated from humans with severe illnesses. Furthermore, most outbreaks of HC and HUS have been attributed to strains of the enterohemorrhagic serotype O157:H7. However, as non-O157 STEC are more prevalent in animals and are food contaminants, humans are probably more exposed to these strains [1-3]. In Spain, STEC represent a significant cause of sporadic cases of human infection [3]. STEC isolates have caused eight outbreaks in Spain: six produced by the serotype O157:H7 (mainly of PT2), one by the serotype O26:H11, and one by the serotype O111:H-[3].

Pathogenicity of STEC is associated with various virulence factors. The main factor is the capacity to form two potent phage-encoded cytotoxins called Shiga-toxins (Stx_1 _and Stx_2_) or verocytotoxins (VT1 and VT2) [1]. In addition to toxin production, another virulence-associated factor expressed by STEC is a protein called intimin encoded by the *eae *gene and responsible for intimate attachment of STEC to the intestinal epithelial cells, and which causes attaching and effacing (A/E) lesions in the intestinal mucosa [4,5]. The association between infections with intimin-positive STEC and severe illnesses in humans was demonstrated previously, but it was also shown that intimin is not essential for the virulence of certain STEC strains. At present, 21 (α1, α2, β1, ξR/β2B, δ/β2O, κ, γ1, γ2, θ, ε1, νR/ε2, ζ, η1, η2, ι1, μR/ι2, λ, μB, νB, ξB and ο) types and subtypes of intimins encoding in the *eae *gene have been identified [5-8]. It is discussed that different intimins may be responsible for different host- and tissue cell tropism [9]. A factor that may also affect virulence of STEC is the enterohemolysin (Ehly), also called enterohemorrhagic *E. coli *haemolysin (EHEC-*HlyA*), encoded by the *ehxA *gene [10].

Ruminants, and especially cattle, represent one of the largest reservoirs of STEC, undercooked minced beef and raw milk being the major vehicles of STEC outbreaks [6,11-15]. The results of our previous studies indicated that STEC colonization is widespread among cattle in Spain. Between 1993 and 1995, 1,069 healthy cattle were examined for STEC colonization. STEC-positive animals were found in 95% of the farms examined and the estimated proportion of positive cattle in each farm ranged from 0 to 100%. The overall prevalence rates of non-O157 STEC colonization were estimated to be 37% in calves and 27% in cows. In a survey between 1998 and 1999, STEC O157:H7 was isolated from 55 (12%) of 471 feedlot calves (four to eight months of age). Although only a mean of three animals per herd were sampled, STEC O157:H7 isolates were detected in 32 (22%) of the 145 feedlots examined [11,13].

Our current investigation is the first large study in Spain on prevalence of STEC O157 and non-O157 in raw beef products. In view of the increasing importance of STEC as emerging food-borne pathogens, the high STEC prevalence in cattle and the lack of data from STEC isolated from food in Spain, the objective of this study were to determine the prevalence of STEC O157:H7 and non-O157 in a large sampling of minced beef collected from 30 local stores in Lugo city between 1995 and 2003. Another objective was to establish the serotypes, virulence genes and intimin types of those STEC isolated from minced beef to determine their potential pathogenicity to humans.

## Results and discussion

In the present study STEC were detected in 95 (12%) of the 785 minced beef samples collected. STEC O157:H7 was isolated from eight (1.0%) samples and non-O157 STEC were isolated from 90 (11%) (Table 1). In three samples, both STEC O157:H7 and non-O157 (O8:H21, O22:H8, O110:H-) were isolated. The best results for detection and isolation of STEC were obtained when the cells were plated onto cefixime tellurite sorbitol MacConkey (CTSMAC) agar (18 h at 44°C) and MacConkey (MAC) agar (18 h at 44°C). The highest prevalence of STEC was detected in the samples with higher level of fecal contamination. Thus, STEC were detected in 33% of minced beef samples with more than 999 *E. coli *per gramme (Table 2).

The comparison of data on STEC prevalence in food is subject to certain limitations. First, the use of different sampling methods on different types of products. Second, protocols for isolation and detection of these pathogens vary and are not uniform among and within laboratories. Third, most studies are focused on O157:H7, and thus underestimate STEC prevalence by ignoring the presence of non-O157 STEC. In any case, figures found in our laboratory do not differ greatly from those reported abroad (12% in New Zealand, 11% in Canada, 11% in the United Kingdom, 9% in Thailand) with studies carried out on the prevalence of both O157 and non-O157 STEC in minced beef [16-19]. Higher prevalences were reported in other countries (i.e. USA, Argentina, India). Thus, three main studies were carried out in Washington state with prevalences of 23% [20], 17% [21], and a quite different 1% [22]. In Argentina, Parma *et al. *[23] detected a prevalence of 30% in 50 minced beef samples. In India, Khan *et al. *[24] found 50% of 111 minced beef samples positive for *stx *by PCR.

In our study STEC O157:H7 was isolated from eight (1.0%) samples. A similar prevalence was detected in different countries: 1% and 0.4% in United Kingdom [25,26]; 1% in The Netherlands [27]; 0.4% and 2% in Italy [28,29]; 0.3% in Czeck Republic [30]. In USA, prevalences reported varied from 0% [31], 0.7% [32] and 2.8% [33]. Higher prevalences were detected in Argentina 3.8% [34], China 5% [35], Perú 19% [36], and in Canada, with two studies of 2% and 29% of prevalence, respectively [32,37].

Analyzing Table 1, we can appreciate a longitudinal maintenance of the prevalence of non-O157 STEC in minced beef samples, with a decline prevalence occurred during 2002. However, it should be considered that only 20 samples were analysed during this year. The same happens with STEC O157:H7, whose prevalence fluctuates from 0% to 1.3%, with the exception of 1995, when it increased to 5%. The present study comprises data from 1995 to 2003, and data from this longitudinal period does not reflect any control measures to reduce contamination in raw minced beef sold in different stores from Lugo.

STEC strains that cause human infections belong to a large number of O:H serotypes (a total of 472 serotypes are listed in the authors' website [38]. Most outbreaks of HC and HUS have been attributed to strains of the enterohemorrhagic serotype O157:H7 [39]. However, as Non-O157 STEC are more prevalent in animals and as contaminant in foods, humans are probably more exposed to these strains. Infections with some non-O157 STEC types, such as O26:H11 or H-(non-motile), O91:H21 or H-, O103:H2, O111:H-, O113:H21, O117:H7, O118:H16, O121:H19, O128:H2 or H-, O145:H28 or H- and O146:H21 are frequently associated with severe illnesses in humans, but the role of other non-O157 STEC types in human illnesses needs further examination [2,3].

In the present study we have characterized 96 STEC isolates (Table 3). These isolates belonged to 42 O serogroups, 61 O:H serotypes (including 10 new serotypes). The majority (68%) of STEC isolates belonged to serotypes previously found in human STEC and 38% to serotypes (O5:H-, O8:H21, O22:H8, O26:H11, O26:H-, O76:H7, O91:H-, O103:H2, O103:H-, O111:H-, O112:H2, O113:H21, O118:H16, O145:H-, O157:H7, O174:H21, O174:H-, ONT:H4 and ONT:H-) (ONT = O not typeable) associated with STEC isolated from patients with HUS. By PCR the 96 STEC isolates showed that 28 (29%) carried *stx*_1 _genes, 49 (51%) *stx*_2_, and 19 (20%) both *stx*_1 _and *stx*_2_. Enterohemolysin (*ehxA*) and intimin (*eae*) virulence genes were detected in 43 (45%) and 25 (26%) of the isolates, respectively. A total of 71 different seropathotypes (associations between serotypes and virulence genes) were detected, being seropathotype O157:H7 *stx*_1_*stx*_2_*eae*-γ1 *ehxA *the most common (five isolates), followed by O8:H21 *stx*_2 _and ONT:H21 *stx*_1 _(four isolates, respectively), O26:H11 *stx*_1_*eae*-β1 *ehxA*, and O64:H5 *stx*_1 _(three isolates, respectively). Like other authors [40], we have observed that STEC isolated from beef and human STEC of the same serotype have similar known virulence-associated properties.

At least 90 phage types have been reported for STEC O157:H7 [41] but only seven of these (PT2, PT4, PT8, PT14, PT21/28, PT32 and PT54) accounted for the majority (>75%) of the human strains isolated in Europe and Canada [39]. Phage types PT2 and PT8 were predominant in human STEC O157:H7 strains in Spain as well as in many other European countries, including Belgium, Finland, Germany, Italy, England and Scotland; whereas PT14 was the most frequently isolated in Canada. Six different phage types were detected among the eight O157:H7 strains isolated in this study: PT2 and PT8 (two isolates, respectively); PT21/28, PT32, PT54, PT atypical (one isolate, each). Interestingly, seven of those eight isolates belonged to the most common phage types associated with severe human illnesses in Europe and Canada [39].

The *eae *gene, which has been shown to be necessary for attaching and effacing activity, encodes a 94- to 97-kDa outer membrane protein (OMP) which is termed intimin [4]. Numerous investigators have underlined the strong association between the carriage of *eae *gene and the capacity of STEC strains to cause severe human illnesses, especially HUS [1-3]. This important virulence gene was detected in 100% of STEC O157:H7 and in 19% of non-O157 isolates from beef in the present study. Nevertheless, production of intimin is not essential for pathogenesis, because a number of sporadic cases of HUS have been caused by *eae*-negative non-O157 STEC strains. Thus, STEC O104:H21 and O113:H21 strains lacking *eae *gene were responsible for an outbreak and a cluster of three HUS cases in USA and Australia, respectively [1,42].

Differentiation of intimin alleles represents an important tool for STEC typing in routine diagnostics as well as in pathogenesis, epidemiological, clonal and immunological studies [5,43-48]. The C-terminal end of intimin is responsible for receptor binding, and it has been suggested that different intimins may be responsible for different host tissue cell tropism. The 5'regions of *eae *genes are conserved, whereas the 3'regions are heterogeneous. This observation led to the construction of universal PCR primers and allele-specific PCR primers, which has made possible to differentiate at present 21 variants of the *eae *gene encoding 21 different intimin types and subtypes (α1, α2, β1, ξR/β2B, δ/β2O, κ, γ1, γ2, θ, ε1, νR/ε2, ζ, η1, η2, ι1, μR/ι2, λ, μB, νB, ξB and ο) [5,8,48]. In this study, four *eae *variants were differentiated: γ1 (in nine isolates), β1 (eight isolates), ε1 (three isolates), and θ (two isolates).

## Conclusion

Seropathotypes O26:H11 *stx*_1 _*eae*-β1, O157:H7 *stx*_1 _*stx*_2 _*eae*-γ1 and O157:H7 *stx*_2 _*eae*-γ1 are the most frequently observed in STEC that cause human infections in Spain [3]. These highly virulent seropathotypes were detected in 10 of 96 STEC isolates characterized in the present study, confirming minced beef as potential source of STEC isolates pathogenic for humans and reinforces the need of HACCP (Hazard Analysis and Critical Control Point) programs all through food chain, from abattoirs to markets, in order to reduce contamination and consequently, illnesses related to minced beef. In addition, consumers should reduce their risk for STEC foodborne illnesses by following safe food-handling recommendations and by avoiding consumption of raw or undercooked meat products.

## Methods

### Sample collection, culture, and STEC screening

A total of 785 of samples of minced beef were collected from 30 local stores in Lugo city (Spain) between 1995 and 2003. A maximum of 10 samples for week were obtained at random from 10 markets located in different districts. Some stores from those 30 were sampled along the seven years, while others only during a period of the study. In most cases, stores had different meat suppliers.

All minced beef samples were packaged individually in sterile plastic bags, refrigerated for transportation to the laboratory, and processed within 2 h of collection. For isolation of STEC O157:H7 a 25 g portion of each minced beef sample was homogenized in a stomacher with 225 ml of buffered peptone water supplemented with vancomycin 8 mg liter ^-1^, cefixime 0.05 mg liter ^-1^, and cefsulodin 10 mg liter ^-1 ^(BPWvcc) and incubated for 6 h at 37°C. One millilitre of this enriched culture was added to 20 μl of magnetic beads coated with O157-antibodies (Dynabeads anti-*E. coli *O157, Dynal, Oslo) and the immunomagnetic separation (IMS) was performed according to the manufacturer's instructions. The concentrated target cells were plated onto sorbitol MacConkey (SMAC) agar and on cefixime tellurite sorbitol MacConkey (CTSMAC) agar (18 h at 37°C). For isolation of non-O157 STEC a 25 g portion of each minced beef sample was homogenized in an stomacher with 225 ml of buffered peptone water without antibiotics (BPW) and incubated for 6 h at 37°C. The cells were plated onto CTSMAC and MacConkey (MAC) agar (18 h at 37°C and 44°C).

STEC were detected by PCR using specific primers for amplification of *stx*_1_, *stx*_2_, and *eae *genes (Table 4). For PCR, a loopful of bacterial growth taken from the first streaking area of the culture plates was suspended in 0.5 ml of sterile distilled water and boiled for 5 min to release the DNA. For each PCR-positive culture, 10 *E. coli*-like colonies obtained from MAC, SMAC and/or CTSMAC plates were analysed by PCR in order to obtain the STEC isolates for further characterization. If no positive single colony was found among the first 10 colonies, at least 40 more were tested. If still none of the assayed coliform colonies was positive in the PCR reaction, the sample was reported as PCR positive without STEC isolation. Production of Shiga toxins (verocytotoxins) by PCR-positive isolates was confirmed by cytotoxicity tests on Vero and HeLa cells [13].

All STEC isolates were subsequently characterized biochemically with the API 20E system (bioMerieux, France) and serotyped. In samples from which all isolates were identical with respect to the profile of virulence genes and O:H serotypes, only one colony was selected. When one sample yielded colonies with different virulence genes or O:H serotypes, one of each was selected for further characterization.

### Enumeration of *E. coli *(MPN)

Most probable number (MPN) determination was performed by standard method using Petrifilm™ Select E. coli Count Plates (3M Microbiology Products, USA), according to manufacturer's instructions.

### Detection of virulence genes and typing of eae (intimin) genes by PCR

The methodology used for the detection of virulence genes and typing of intimin genes into eae α1, α2, β1, ξR/β2B, δ/β2O, κ, γ1, γ2, θ, ε1, νR/ε2, ζ, η1, η2, ι1, μR/ι2, λ, μB, νB, ξB and ο has been described elsewhere [7,8,12,13,48]. Base sequences and predicted sizes of amplified products for the specific oligonucleotide primers used in this study are shown in Table 4. The oligonucleotide primers were designed by us according to the nucleotide sequences of the virulence genes. Isolates positive for *eae *gene with EAE-1 and EAE-2 primers were further analysed with all different variant primers. Due to the high sequence similarity, specific primers could not be designed for distinguishing between *eae*-γ2 and *eae-θ *genes, *eae-δ *and *eae-κ*, or *eae*-η1 and *eae*-η2. In those cases it was necessary to establish the nucleotide sequence of a fragment from the 3' variable region of the *eae *gene. The nucleotide sequence of the amplification products purified with a QIAquick DNA purification kit (Qiagen) was determined by the dideoxynucleotide triphosphate chain termination method of Sanger, with the BigDye Terminator v3.1 Cycle Sequencing Kit and an ABI 3100 Genetic Analyzer (Applied Bio-Systems).

### Serotyping

The determination of O and H antigens was carried out by the method described by Guinιe *et al. *[49] employing all available O (O1-O185) and H (H1-H56) antisera. All antisera were obtained and absorbed with the corresponding cross-reacting antigens to remove the nonspecific agglutinins. The O antisera were produced in the LREC (Lugo, Spain) and the H antisera were obtained from the Statens Serum Institut (Copenhagen, Denmark).*E. coli *strains representing the new O groups O182 to O185 (unpublished data) were kindly provided by Flemming Scheutz (Statens Serum Institut).

### Phage typing of STEC O157:H7

Phage typing was performed with the method of Khakria *et al. *[41] in the Centro Nacional de Microbiología (Madrid) [39,50] using phages provided by The National Laboratory for Enteric Pathogens, Laboratory for Disease Control, Otawa, Ontario (Canada). The sixteen different phages used were capable of identifying 90 phage types.

### *E. coli *control strains

*E. coli *strains used as controls were: EPEC-2348 (human, O127:H6, *eae*-α1), AEEC-IH2498a (human, O125:H6, *eae*-α2), EPEC-337 (human, O111:H2, *eae*-β1), EPEC-359 (human, O119:H6, *eae*-ξR/β2B), EPEC-BL152.1 (human, O86:H34, *eae*-δ/β2O), AEEC-6044/95 (human, O118:H5, *eae*-κ), STEC-EDL933 (human, O157:H7, *stx*_1_, *stx*_2_, *eae*-γ1), STEC-TW07926 (human, O111:H8, *stx*_1_, *stx*_2_, *eae*-θ), STEC-VTB-286 (bovine, O103:H2, *stx*_1, _*eae*-ε1), AEEC-IH3205a (human, O123:H19, *eae*-νR/ε2), STEC-VTO-50 (ovine, O156:H-, *stx*_1, _*eae*-ζ), AEEC-CF11201 (human, O125:H-, *eae*-η1), H03/53199a (human, ONT:H45, *eae*-η2), AEEC-7476/96 (human, O145:H4, *eae*-ι1), AEEC-217-2 (human, O101:H-, *eae*-μR/ι2), AEEC-68-4 (human, O34:H-, *eae*-λ), EPEC-373 (human, O55:H51, *eae*-μB), AEEC-IH1229a (human, O10:H-, *eae*-νB), STEC-B49 (bovine, O80:H-, *stx*_1_,*eae*-ξB), AEEC-IH2997f (human, O129:H-, *eae*-ο) and K12-185 (negative for *stx*_1_, *stx*_2_, *bfpA*, and *eae *genes). Strains were stored at room temperature in nutrient broth with 0.75% of agar.

## Authors' contributions

AM, CL, and PJ carried out the isolation of the *E. coli *and performed the detection of virulence genes. MB, EAG and GD carried out the typing of the *eae *gene. JEB, MPA and MIB performed the serotyping. AE carried out the phage typing. JB carried out the design of the study and, together with AM, drafted the manuscript. All authors read and approved the final manuscript.

## Figures and Tables

**Table 1:** Prevalence of STEC in minced beef in Spain, from 1995 through 2003

Year	No. of samples analysed	STEC O157:H7		Non-O157 STEC		Total STEC	
1995	58	3	5%	8	14%	10	17%
1996	91	0	0%	8	9%	8	9%
1997	173	1	0.6%	20	12%	21	12%
1998	133	1	0.8%	18	14%	18	14%
2001	80	1	1.3%	6	8%	7	9%
2002	20	0	0%	1	5%	1	5%
2003	230	2	0.9%	29	13%	30	13%

Total	785	8	1.0%	90	11%	95	12%

**Table 2:** Most probable number of *E. coli *and detection of STEC

MPN^a ^of *E. coli*	No. of samples analysed	STEC O157:H7	Non-O157 STEC	Total STEC
<10	488	2 (0.4%)	43 (9%)	43 (9%)
10–99	183	2 (1%)	27 (15%)	29 (16%)
100–999	71	2 (3%)	8 (11%)	9 (13%)
>999	43	2 (5%)	12 (28%)	14 (33%)

^a^Most probable number of *E. coli *per gram of minced beef.

**Table 3:** Seropathotypes (serotypes and virulence genes) of STEC isolates (n = 96).

Serotype	Total	*stx*_1_	*stx*_2_	*eae*	*ehxA*	Serotype	Total	*stx*_1_	*stx*_2_	*eae*	*ehxA*
O1:H20^a^	1	+	-	+ (NR)^d^	+	O112:H2^b^	1	+	+	-	+
O1:H20^a^	1	+	-	-	+	O113:H21^b^	1	-	+	-	+
O2:H8^c^	1	-	+	-	-	O113:H21^b^	1	-	+	-	-
O2:H27^a^	1	-	+	-	-	O116:H16^c^	1	-	+	-	-
O2:H32	1	-	+	-	-	O116:H21^a^	1	+	+	-	+
O2:H-^a^	1	-	+	-	-	O118:H16^b^	1	+	-	+ (β1)	+
O4:H4	1	-	+	-	-	O120:H10^c^	1	-	+	-	-
O5:H-^b^	1	+	+	+ (β1)	+	O128:H31^a^	1	+	-	-	-
O6:H10	1	+	-	-	-	O141:H19	1	-	+	-	-
O6:H49^a^	1	-	+	-	-	O145:H-^b^	1	+	-	+ (γ1)	+
O8:H21^b^	4	-	+	-	-	O146:H21^a^	1	-	+	-	+
O8:H-^a^	2	+	+	-	+	O146:H-^a^	1	+	+	-	+
O15:H16	1	-	+	+ (β1)	-	O156:H8	1	-	+	+ (NR)	-
O15:H18^c^	1	-	+	-	-	O157:H7^b^	1	+	-	+ (γ1)	+
O17:H45	1	-	+	-	-	O157:H7^b^	2	-	+	+ (γ1)	+
O20:H2^c^	1	+	-	-	-	O157:H7^b^	5	+	+	+ (γ1)	+
O21:H21	1	-	+	-	-	O162:H10	1	+	+	-	-
O22:H8^b^	2	-	+	-	-	O166:H28^a^	1	+	+	-	+
O22:H8^b^	2	+	+	-	+	O167:H2	1	+	-	-	-
O26:H11^b^	3	+	-	+ (β1)	+	O168:H8	1	-	+	-	-
O26:H-^b^	1	-	+	-	-	O171:H2^a^	2	-	+	-	-
O26:H42	1	+	-	+ (β1)	+	O174:H10	1	+	+	+ (β1)	-
O39:H21	1	-	+	-	+	O174:H21^b^	1	-	+	-	-
O42:H21^c^	1	-	+	-	-	O174:H-^b^	2	-	+	-	-
O54:H-^c^	1	-	+	-	-	OX178:H16^c^	1	-	+	-	-
O64:H5^c^	3	+	-	-	-	ONT:H4^b^	1	-	+	-	-
O75:H8^a^	1	+	-	-	+	ONT:H19^a^	1	-	+	-	+
O75:H8^a^	1	-	+	-	+	ONT:H21^a^	1	+	+	-	+
O76:H7^b^	1	-	+	+ (NR)	+	ONT:H21^a^	1	+	+	-	-
O77:H41^a^	2	-	+	-	+	ONT:H21^a^	4	+	-	-	-
O88:H8	1	-	+	-	-	ONT:H21^a^	2	-	+	-	-
O91:H-^b^	1	+	+	-	+	ONT:H38	1	-	+	-	-
O103:H2^b^	2	+	-	+ (ε1)	+	ONT:H?	1	-	+	-	+
O103:H-^b^	1	+	-	+ (ε1)	+	ONT:H-^b^	1	+	-	-	-
O110:H-^c^	1	+	-	-	+	ONT:H-^b^	1	-	+	-	-
O111:H-^b^	2	+	-	+ (θ)	+	ONT:H-^b^	1	-	+	-	-

^a ^Serotypes previously found in human STEC.

^b ^Serotypes previously associated with human STEC strains that cause hemolytic uremic syndrome (HUS).

^c ^New serotypes in STEC not reported in previous studies.

^d^Typing of *eae *(intimin) genes was carried out years after the isolation of the strains. Some of them had lost the viability, so *eae *typing could not be carried out (NR).

**Table 4:** PCR primers and conditions for amplification of STEC virulence genes and *eae *typing

Gene	Primer	Oligonucleotide sequence (5'-3')	Fragment size (bp)	Annealing temperature	Primer Coordinates^a^	Accession number
*stx1*	VT1-A	CGCTGAATGTCATTCGCTCTGC	302	55°C	113–134	M17358
	VT1-B	CGTGGTATAGCTACTGTCACC			394–414	
*stx*2	VT2-A	CTTCGGTATCCTATTCCCGG	516	55°C	50–69	M59432
	VT2-B	CTGCTGTGACAGTGACAAAACGC			543–565	
*ehxA*	HlyA1	GGTGCAGCAGAAAAAGTTGTAG	1.551	60°C	238–259	X79839
	HlyA4	TCTCGCCTGATAGTGTTTGGTA			1767–1788	
*eae*^b^	EAE-1	GGAACGGCAGAGGTTAATCTGCAG	346	55°C	631–654	AF022236
	EAE-2	GGCGCTCATCATAGTCTTTC			957–976	
*eae*-α1	EAE-FB	AAAACCGCGGAGATGACTTC	820	60°C	1909–1928	AF022236
	EAE-A	CACTCTTCGCATCTTGAGCT			2709–2728	
*eae*-α2	IH2498aF	AGACCTTAGGTACATTAAGTAAGC	517	60°C	2099–2122	AF530555
	IH2498aR	TCCTGAGAAGAGGGTAATC			2597–2615	
*eae*-β1	B1F	CACAATTAATGCACCGGGT	241	55°C	2499–2517	AF453441
	B1R	GCTTGATACACCTGATGACT			2720–2739	
*eae*-ξR/β2B	B2F	TGAAGGGGGGAACCCCTGTG	564	62°C	2054–2073	AF530556
	B2R	TTTCTTTTGACTGTGCTAAAGC			2596–2617	AJ715407
*eae*-δ/κ/β2O	EAE-FB	AAAACCGCGGAGATGACTTC	830	60°C	1909–1928	U66102
	EAE-D	CTTGATACACCCGATGGTAAC			2718–2738	
*eae*-γ1	EAE-FB	AAAACCGCGGAGATGACTTC	804	60°C	1909–1928	AF071034
	EAE-C1	AGAACGCTGCTCACTAGATGTC			2691–2712	
*eae*-θ/γ2	EAE-C2F	AGAACGTTACTGGTGACTTA	414	58°C	2303–2322	AF025311
	EAE-C2R	CTGATATTTTATCAGCTTCA			2697–2716	
*eae*-ε1	EAE-FB	AAAACCGCGGAGATGACTTC	722	66°C	1909–1928	AF116899
	LP5	AGCTCACTCGTAGATGACGGCAAGCG			2605–2630	
*eae*-νR/ε2	EAE-E2F	AATACAGAAGTTAAGGCAT	378	58°C	2230–2248	AF530554
	EAE-E2R	ACGACCACTATTCATTTC			2590–2607	
*eae*-ζ	Z1	GGTAAGCCGTTATCTGCC	206	62°C	2062–2079	AF449417
	Z2	ATAGCAAGTGGGGTGAAG			2250–2267	
*eae*-η1/η2	EAE-FB	AAAACCGCGGAGATGACTTC	702	60°C	1899–1918	AJ308550
	ETA-B	TAAGCGACCACTATTCGTG			2582–2600	
*eae*-η1	ETA-FN	CGCTTTGTTTAATGCCGATAAA	410	62°C	1074–1095	AJ308550
	ETA-RN	GACTGCGTAATGCACTG			1467–1483	
*eae*-ι1	EAE-FB	AAAACCGCGGAGATGACTTC	651	55°C	1909–1928	AJ308551
	IOTA-B	GTCATATTTAACTTTTACACTA			2538–2559	
*eae*-μR/ι2,	Iota2-F	CTGGTAAAGCGATAGTCAAAC	936	58°C	1850–1870	AF530553
	Iota2-R	GCGTTTTTGAAGAAACATTTTGC			2763–2785	
*eae*-λ	68.4F	CGGTCAGCCTGTGAAGGGC	370	64°C	2061–2079	AJ715409
	68.4R	AATACCGGAAGAGGCATCTAT			2410–2430	
*eae*-μB	EAE-FB	AAAACCGCGGAGATGACTTC	655	60°C	1909–1928	AJ705049
	FV373R	ACTCATCATAATAAGCTTTTTGG			2541–2563	
*eae*-νB	IH1229aF	CACAGCTTACAATTGATAACA	311	60°C	2255–2275	AJ705050
	IH1229aR	CTCACTATAAGTCATACGACT			2545–2565	
*eae*-ξB	EAE-FB	AAAACCGCGGAGATGACTTC	468	66°C	1909–1928	AJ705051
	B49R	ACCACCTTTAGCAGTCAATTTG			2355–2376	
*eae*-ο	H2997fF	AGCGTTAGCAATGCCGCAGTTGAT	271	60°C	2203–2226	AJ876647
	IH2997fR	CAACGGTAATTGTTGTTTCC			2454–2473	AJ876648

^a ^Location within eae gene. The oligonucleotide primers were designed by us according to the nucleotide sequences of the virulence genes.

^b^Universal oligonucleotide primer pair EAE1 and EAE-2 with homology to the 5'conserved region of *eae *gene (detects all types of *eae *variants described at the moment). Isolates positive for *eae *gene with EAE-1 and EAE-2 primers were further analysed with all different variant primers.

## References

[B1] Paton JC, Paton AW (1998). Pathogenesis and diagnosis of shiga toxin-producing *Escherichia coli *infections. Clin Microbiol Rev.

[B2] Beutin L, Krause G, Zimmermann S, Kaulfuss S, Gleier K (2004). Charaterization of Shiga toxin-producing *Escherichia coli *strains isolated from human patients in Germany over a 3-year period. J Clin Microbiol.

[B3] Blanco JE, Blanco M, Alonso MP, Mora A, Dahbi G, Coira MA, Blanco J (2004). Serotypes, virulence genes and intimin types of Shiga toxin (verotoxin)-producing *Escherichia coli *isolates from human patients: prevalence in Lugo, Spain, from 1992 through 1999. J Clin Microbiol.

[B4] Kaper JB, Elliott S, Sperandio V, Perna NT, Mayhew GF, Blattner FR, Kaper JB, O'Brien AD (1998). Attaching and effacing intestinal histopathology and the locus of enterocyte effacement. Escherichia coli O157:H7 and other Shiga toxin-producing E coli strains.

[B5] Garrido P, Blanco M, Moreno-Paz M, Briones C, Dahbi G, Blanco JE, Blanco J, Parro V (2006). STEC-EPEC oligonucleotide microarray: a new tool for typing genetic variants of the LEE pathogenicity island of human and animal Shiga toxin-producing *Escherichia coli *(STEC) and enteropathogenic *E. coli *(EPEC) strains. Clin Chem.

[B6] Blanco M, Podola NL, Krüger A, Sanz ME, Blanco JE, González EA, Dahbi G, Mora A, Bernárdez MI, Etcheverria AL, Arroyo GH, Lucchesi PMA, Parma AE, Blanco J (2004). Virulence genes and intimin types of Shiga-toxin-producing *Escherichia coli *isolated from cattle and beef products in Argentina. Int Microbiol.

[B7] Blanco M, Schumacher S, Tasara T, Zweifel C, Blanco JE, Dahbi G, Blanco J, Stephan R (2005). Serotypes, intimin variants and other virulence factors of *eae *positive *Escherichia coli *strains isolated from healthy cattle in Switzerland. Identification of a new intimin variant gene (eae-η2). BMC Microbiol.

[B8] Blanco M, Blanco JE, Dahbi G, Alonso MP, Mora A, Coira MA, Madrid C, Juárez A, Bernárdez MI, González EA, Blanco J (2006). Identification of two new intimin types in atypical enteropathogenic *Escherichia coli*. Int Microbiol.

[B9] Torres AG, Zhou G, Kaper JB (2005). Adherence of diarrheagenic *Escherichia coli *strains to epithelial cells. Infect Immun.

[B10] Schmidt H, Beutin L, Karch H (1995). Molecular analysis of the plasmid-encoded hemolysin of *Escherichia coli *O157:H7 strain EDL 933. Infect Immun.

[B11] Blanco J, Blanco M, Blanco JE, Mora A, Alonso MP, González EA, Bernárdez MI, Duffy G, Garvey P, McDowell D (2001). Epidemiology of verocytotoxigenic *Escherichia coli *(VTEC) in ruminants. Verocytotoxigenic Escherichia coli.

[B12] Blanco M, Blanco JE, Mora A, Rey J, Alonso JM, Hermoso M, Hermoso J, Alonso MP, Dhabi G, González EA, Bernárdez MI, Blanco J (2003). Serotypes, virulence genes, and intimin types of Shiga toxin (verotoxin)-producing *Escherichia coli *isolates from healthy sheep in Spain. J Clin Microbiol.

[B13] Blanco M, Blanco JE, Mora A, Dahbi G, Alonso MP, González EA, Bernárdez MI, Blanco J (2004). Serotypes, virulence genes, and intimin types of Shiga toxin (Verotoxin)-producing *Escherichia coli *isolates from cattle in Spain and identification of a new intimin variant gene (eae-ξ). J Clin Microbiol.

[B14] Zweifel C, Blanco JE, Blanco M, Blanco J, Stephan R (2004). Serotypes and virulence genes of ovine non-O157 Shiga toxin-producing Escherichia coli in Switzerland. Int J Food Microbiol.

[B15] Zweifel C, Schumacher S, Blanco M, Blanco JE, Tasara T, Blanco J, Stephan R (2005). Phenotypic and genotypic characteristics of non-O157 Shiga toxin-producing *Escherichia coli *(STEC) from Swiss cattle. Vet Microbiol.

[B16] Read SC, Gyles CL, Clarke RC, Lior H, McEwen S (1990). Prevalence of verocytotoxigenic *Escherichia coli *in ground beef, pork, and chicken in southwestern Ontario. Epidemiol Infect.

[B17] Suthienkul O, Brown E, Seriwatana J, Tienthongdee S, Sastravaha S, Echeverria P (1990). Shiga-like-toxin-producing *Escherichia coli *in retail meats and cattle in Thailand. Appl Environ Microbiol.

[B18] Willshaw GA, Smith HR, Roberts D, Thirlwell J, Cheasly T, Rowe B (1993). Examination of raw beef products for the presence of vero cytotoxin producing *Escherichia coli*, particularly those of serogroup O157. J Appl Bacteriol.

[B19] Brooks HJL, Mollison BD, Bettelheim KA, Matejka K, Paterson KA, Wark VK (2001). Occurrence and virulence factors of non-O157 Shiga toxin-producing *Escherichia coli *in retail meat in Dunedin, New Zealand. Lett Appl Microbiol.

[B20] Samadpour M, Ongerth JE, Liston J, Tran N, Nguyen D, Whittam TS, Wilson RA, Tarr PI (1994). Occurrence of Shiga-like toxin-producing *Escherichia coli *in retail fresh seafood, beef, lamb, pork, and poultry from grocery stores in Seattle, Washington. Appl Environ Microbiol.

[B21] Samadpour M, Kubler M, Buck FC, Depavia GA, Mazengia E, Stewart J, Yang P, Alfi D (2002). Prevalence of Shiga toxin-producing *Escherichia coli *in ground beef and cattle feces from King County, Washington. J Food Prot.

[B22] Schroeder CM, White DG, Ge B, Zhang Y, McDermott PF, Ayers S, Zhao S, Meng J (2003). Isolation of antimicrobial-resistant *Escherichia coli *from retail meats purchased in Greater Washington, DC, USA. Int J Food Microbiol.

[B23] Parma AE, Sanz ME, Blanco JE, Blanco J, Viñas MR, Blanco M (2000). Virulence genotypes and serotypes of verotoxigenic *Escherichia coli *isolated from cattle and foods in Argentina. Eur J Epidemiol.

[B24] Khan A, Yamasaki S, Sato T, Ramamurthy T, Pal A, Datta S, Chowdhury NR, Das SC, Sikdar A, Tsukamoto T, Bhattacharya SK, Takeda Y, Nair GG (2002). Prevalence and genetic profiling of virulence determinants of non-O157 Shiga toxin-producing *Escherichia coli *isolated from cattle, beef, and humans, Calcutta, India. Emerg Infect Dis.

[B25] Chapman PA, Siddons CA, Cerdan Malo AT, Harkin MA (2000). A one year study of *Escherichia coli *O157 in raw beef and lamb products. Epidemiol Infect.

[B26] Chapman PA, Cerdán Malo AT, Ellin M, Ashton R, Harkin MA (2001). *Escherichia coli *O157 in cattle and sheep at slaughter, on beef and lamb carcasses and in raw beef and lamb products in South Yorkshire, UK. Int J Food Microbiol.

[B27] Heuvelink AE, Zwartkruis-Nahuis JTM, Beumer RR, De Boer E (1999). Occurrence and survival of verocytotoxin-producing *Escherichia coli *O157 in meats obtained from retail outlets in The Netherlands. J Food Prot.

[B28] Conedera G, Dalvit P, Martín M, Galiero G, Gramaglia M, Goffredo E, Loffredo G, Morabito S, Ottaviani D, Paterlini F, Pezzotti G, Pisanu M, Semprini P, Caprioli A (2004). Verocytotoxin-producing *Escherichia coli *O157 in minced beef and dairy products in Italy. Int J Food Microbiol.

[B29] Stampi S, Caprioli A, De Luca G, Quaglio P, Sacchetti R, Zanetti F (2004). Detection of *Escherichia coli *O157 in bovine meat products in northern Italy. Int J Food Microbiol.

[B30] Lukásová J, Abraham B, Cupáková S (2004). Occurrence of *Escherichia coli *O157 in raw material and food in Czech Republic. J Vet Med B.

[B31] Tarr PI, Tran TN, Wilson RA (1999). *Escherichia coli *O157:H7 in retail ground beef in Seattle: results of a one-year prospective study. J Food Prot.

[B32] Doyle MP, Schoeni JL (1987). Isolation of *Escherichia coli *O157:H7 from retail fresh meats and poultry. Appl Environ Microbiol.

[B33] Padhye NV, Doyle MP (1991). Rapid procedure for detecting Enterohemorrhagic *Escherichia coli *O157:H7 in food. Appl Environ Microbiol.

[B34] Chinen I, Tanaro JD, Miliwebsky E, Lound LH, Chillemi G, Ledri S, Baschkier A, Scarpin M, Manfredi E, Rivas M (2001). Isolation and characterization of *Escherichia coli *O157:H7 from retail meats in Argentina. J Food Prot.

[B35] Zhou Z, Nishikawa Y, Zhu P, Hong S, Hase A, Cheasty T, Smith HR, Zheng M, Haruki K (2002). Isolation and characterization of Shiga toxin-producing *Escherichia coli *O157:H7 from beef, pork, and cattle fecal samples in Changchun, China. J Vet Med Sci.

[B36] Mora A, León SL, Blanco M, Blanco JE, López C, Dahbi G, Echeita A, González EA, Blanco J (2007). Phage types, virulence genes and PFGE profiles of Shiga toxin-producing Escherichia coli O157:H7 isolated from raw beef meat, soft cheese and vegetables in Lima (Peru). Int J Food Microbiol.

[B37] Sekla L, Milley D, Stackiw W (1990). Verotoxin-producing *Escherichia coli *in ground beef in Manitoba. Can Med Assoc J.

[B38] http://www.lugo.usc.es/ecoli/SEROTIPOSHUM.htm.

[B39] Mora A, Blanco M, Blanco JE, Alonso MP, Dahbi G, Thompson-Carter F, Usera MA, Bartolomé R, Prats G, Blanco J (2004). Phage Types and Genotypes of Human and Animal Shiga Toxin-Producing *Escherichia coli *O157:H7 in Spain. Identification of two predominating phage types (PT2 and PT8). J Clin Microbiol.

[B40] Meng J, Zhao S, Doyle MP (1998). Virulence genes of shiga toxin-producing *Escherichia coli *isolated from food, animals and humans. Int J Food Microbiol.

[B41] Khakria R, Duck D, Lior H (1990). Extended phage-typing scheme for *Escherichia coli *O157:H7. Epidemiol Infect.

[B42] Paton AW, Woodrow MC, Doyle RM, Lanser JA, Paton JC (1999). Molecular characterization of a shiga toxigenic *Escherichia coli *O113:H21 strain lacking *eae *responsible for a cluster of cases of hemolytic-uremic syndrome. J Clin Microbiol.

[B43] Adu-Bobie J, Frankel G, Bain C, Goncalves AG, Trabulsi LR, Douce G, Knutton S, Dougan G (1998). Detection of intimins α, β, γ, and δ, four intimin derivatives expressed by attaching and effacing microbial pathogens. J Clin Microbiol.

[B44] Oswald E, Schmidt H, Morabito S, Karch H, Marchès O, Caprioli A (2000). Typing of intimin genes in human and animal enterohemorrhagic and enteropathogenic *Escherichia coli*: characterization of a new intimin variant. Infect Immun.

[B45] Tarr CL, Whittam S (2002). Molecular evolution of the intimin gene in O111 clones of pathogenic *Escherichia coli*. J Bacteriol.

[B46] Zhang WL, Köhler B, Oswald E, Beutin L, Karch H, Morabito S, Caprioli A, Suerbaum S, Schmidt H (2002). Genetic diversity of intimin genes of attaching and effacing *Escherichia coli *strains. J Clin Microbiol.

[B47] Ramachandran V, Brett K, Hornitzky MA, Dowton M, Bettelheim KA, Walker MJ, Djordjevic SP (2003). Distribution of intimin subtypes among *Escherichia coli *isolates from ruminant and human sources. J Clin Microbiol.

[B48] Blanco M, Blanco JE, Dahbi G, Mora A, Alonso MP, Varela G, Gadea MP, Schelotto F, Gonzalez EA, Blanco J (2006). Typing of intimin (eae) genes from enteropathogenic Escherichia coli (EPEC) isolated from children with diarrhoea in Montevideo, Uruguay: identification of two novel intimin variants (μB and ξR/β2B). J Med Microbiol.

[B49] Guinée PAM, Jansen WH, Wadström T, Sellwood R, Leeww PW, Guinée PAM (1981). *Escherichia coli *associated with neonatal diarrhoea in piglets and calves. Laboratory Diagnosis in Neonatal Calf and Pig diarrhoea: Current Topics in Veterinary and Animal Science.

[B50] Mora A, Blanco JE, Blanco M, Alonso MP, Dhabi G, Echeita A, Gonzalez EA, Bernardez MI, Blanco J (2005). Antimicrobial resistance of Shiga toxin (verotoxin)-producing Escherichia coli O157:H7 and non-O157 strains isolated from humans, cattle, sheep and food in Spain. Res Microbiol.

